# Supplementary material for: miR‐467 regulates inflammation and blood insulin and glucose
Source: J Cell Mol Med. 2021 Feb 10;25(5):2549–62. doi: 10.1111/jcmm.16224 (PMC7933977; doi:10.1111/jcmm.16224)
Supplement: Supplementary file 1 — Fig S1‐13 [file JCMM-25-2549-s001.pdf]

Figure S1

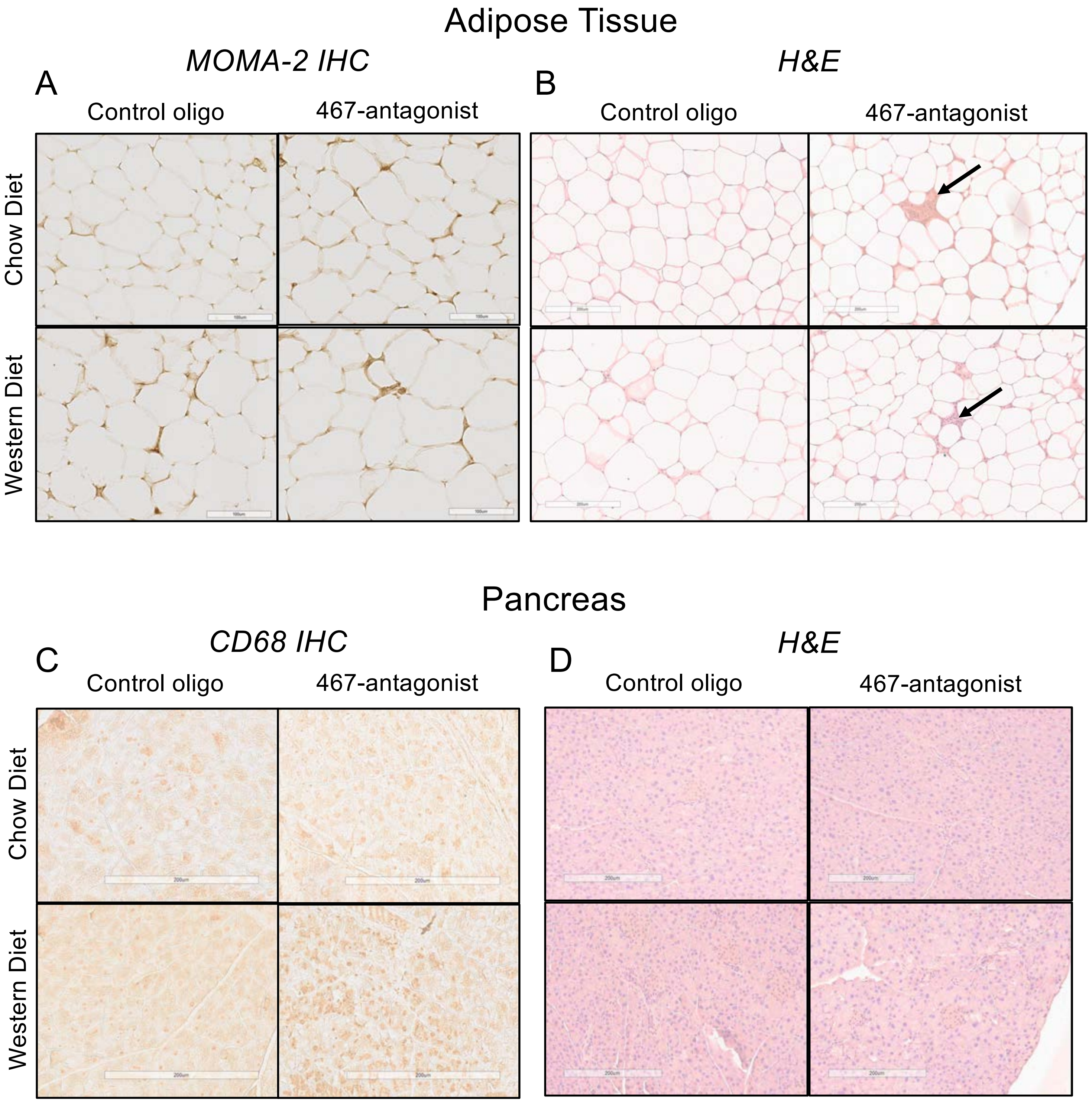
Adipose Tissue
MOMA-2 IHC
A
Control oligo
467-antagonist
Chow Diet
Western Diet
H&E
B
Control oligo
467-antagonist
Pancreas
CD68 IHC
C
Control oligo
467-antagonist
Chow Diet
Western Diet
H&E
D
Control oligo
467-antagonist

Figure S2

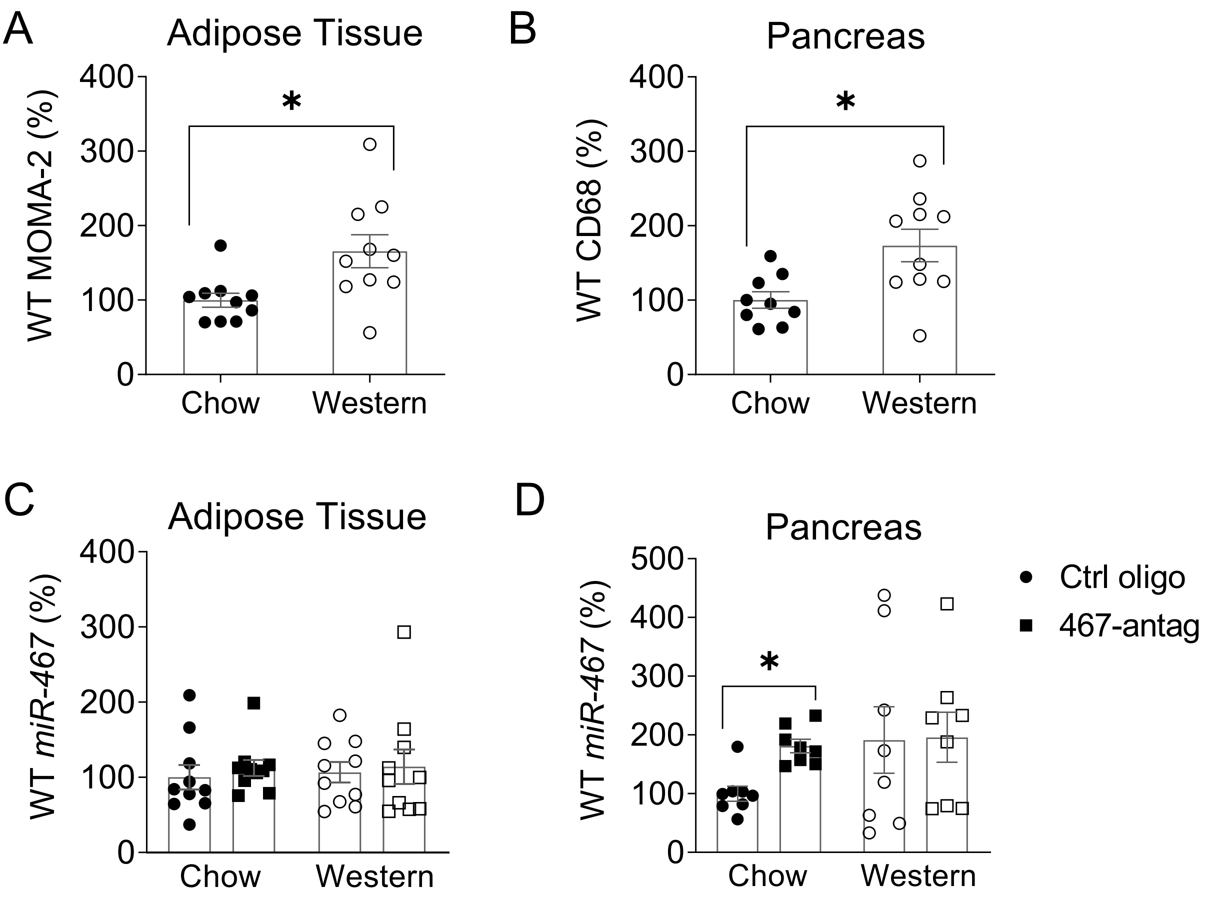

A
Adipose Tissue
WT MOMA-2 (%)
400
300
200
100
0
*
Chow
Western
B
Pancreas
WT CD68 (%)
400
300
200
100
0
*
Chow
Western
C
Adipose Tissue
WT miR-467 (%)
400
300
200
100
0
Chow
Western
D
Pancreas
WT miR-467 (%)
500
400
300
200
100
0
*
Chow
Western
Ctrl oligo
467-antag

Figure S3

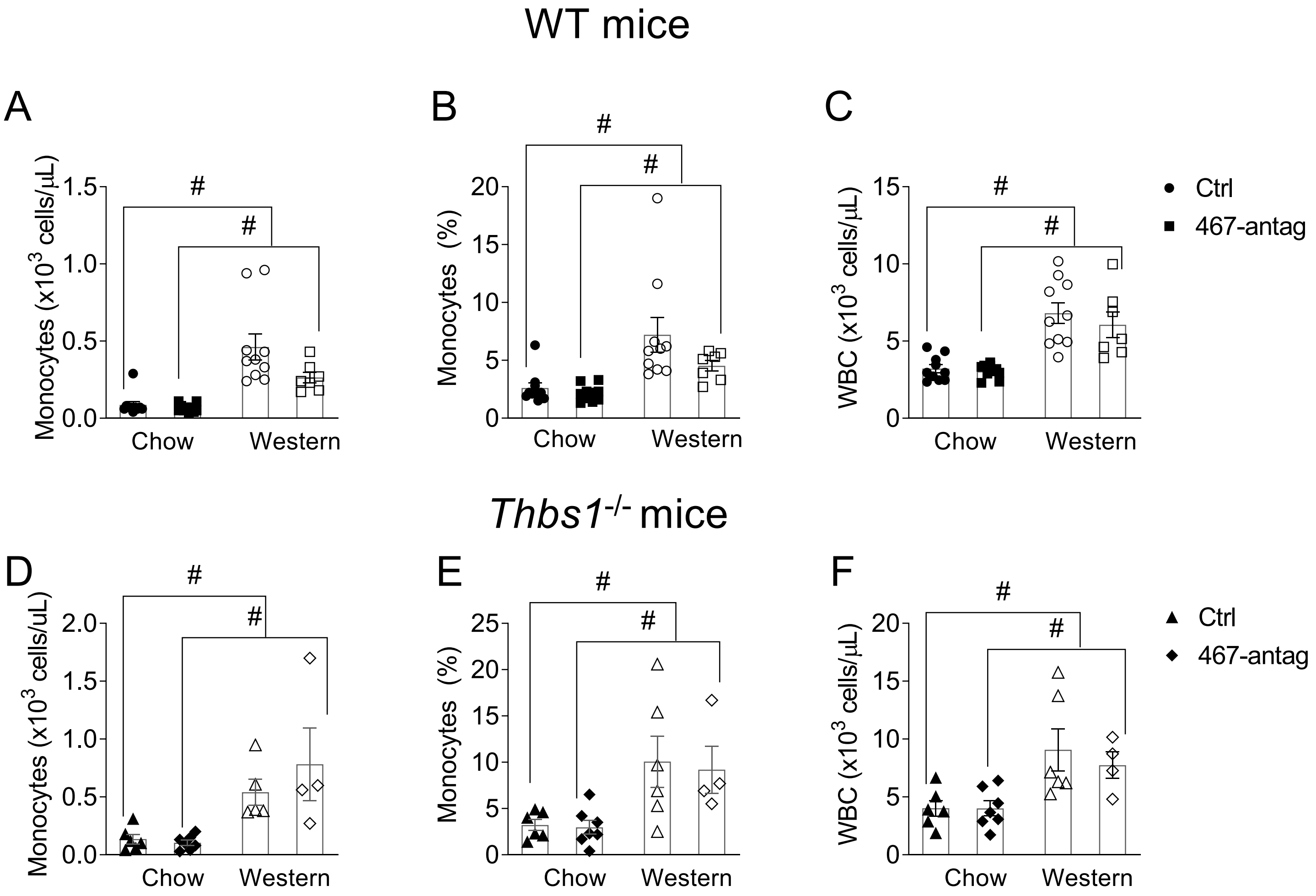
WT mice
A
B
C
Monocytes (x10^3 cells/μL)
Monocytes (%)
WBC (x10^3 cells/μL)
Chow
Western
#
Ctrl
467-antag
Thbs1-/- mice
D
E
F
Monocytes (x10^3 cells/uL)
Monocytes (%)
WBC (x10^3 cells/μL)
Chow
Western
Ctrl
467-antag

Figure S4

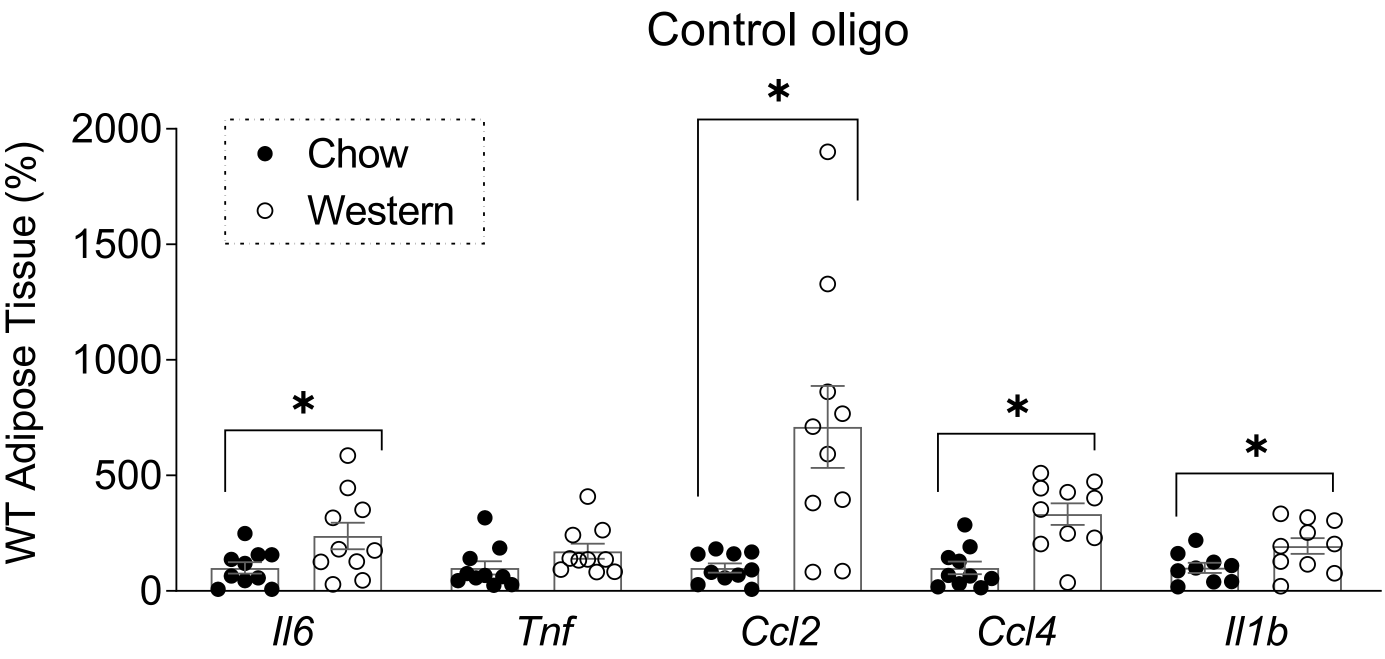
Control oligo
WT Adipose Tissue (%)
2000
1500
1000
500
0
Chow
Western
*
*
*
*
Il6
Tnf
Ccl2
Ccl4
Il1b

Figure S5

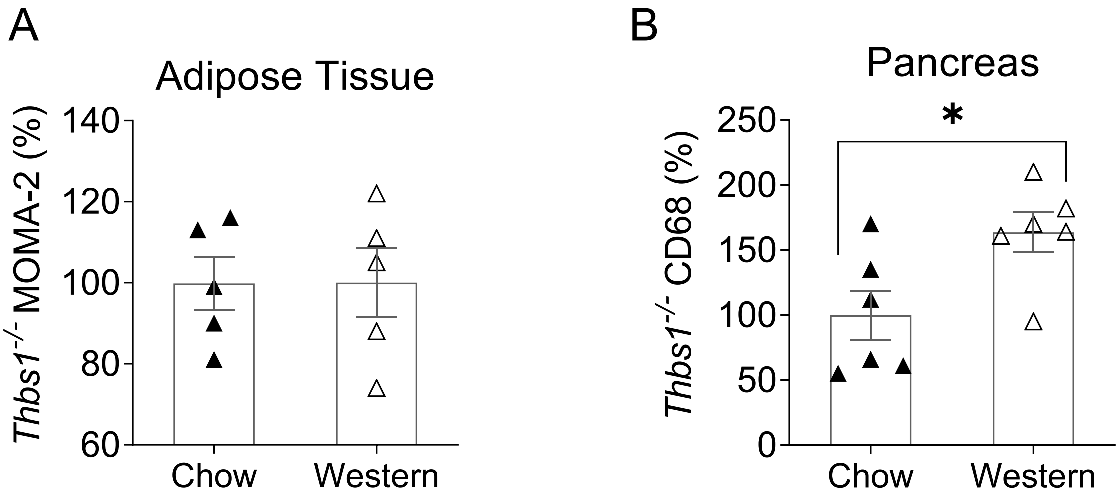
A
Adipose Tissue
Thbs1-/- MOMA-2 (%)
140
120
100
80
60
Chow
Western
B
Pancreas
Thbs1-/- CD68 (%)
*
250
200
150
100
50
0
Chow
Western

Figure S6

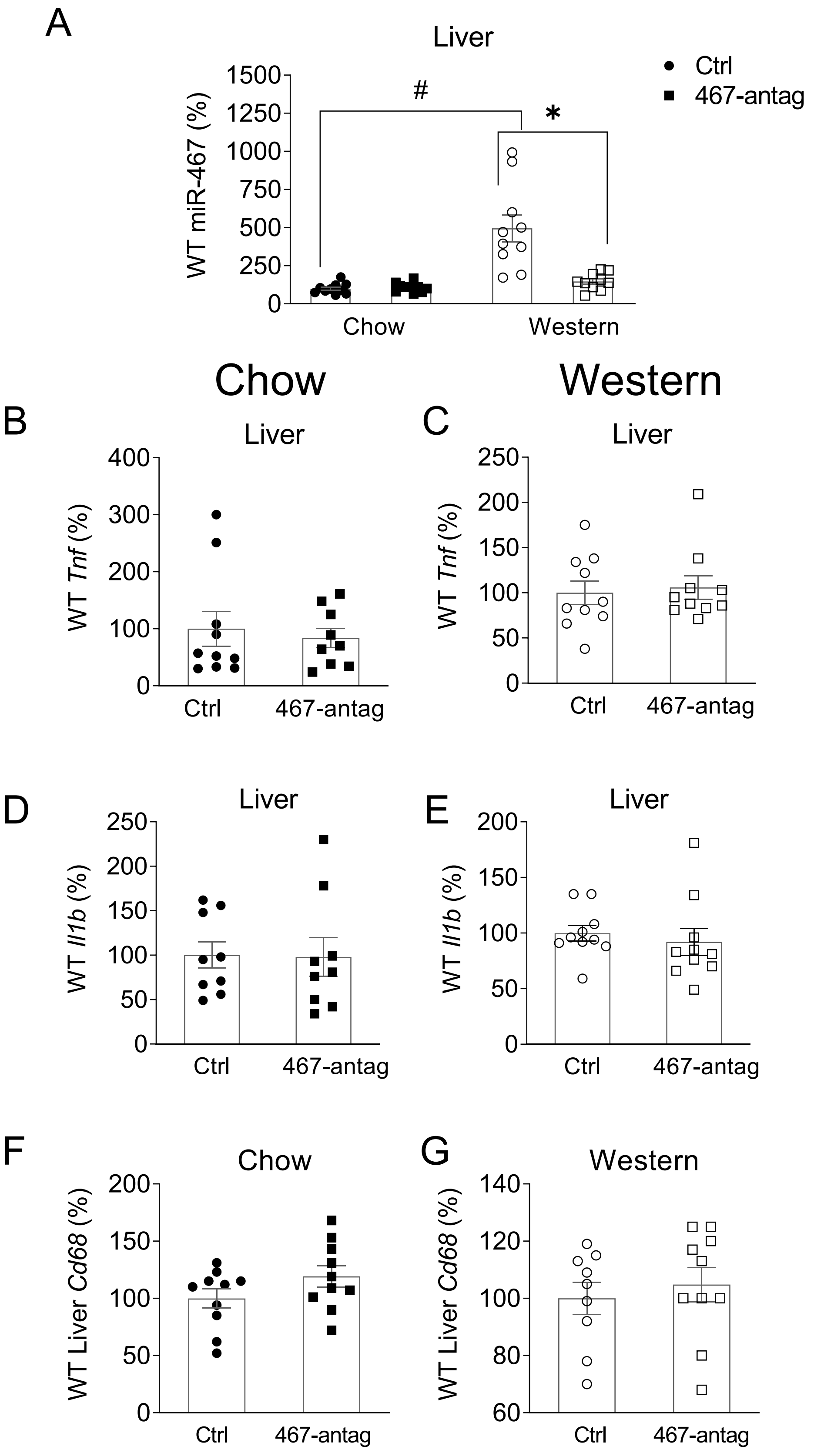

A
Liver
Ctrl
467-antag
WT miR-467 (%)
1500
1250
1000
750
500
250
0
#
*
Chow
Western
Chow
Western
B
Liver
WT Tnf (%)
400
300
200
100
0
Ctrl
467-antag
C
Liver
WT Tnf (%)
250
200
150
100
50
0
Ctrl
467-antag
D
Liver
WT Il1b (%)
250
200
150
100
50
0
Ctrl
467-antag
E
Liver
WT Il1b (%)
200
150
100
50
0
Ctrl
467-antag
F
Chow
WT Liver Cd68 (%)
200
150
100
50
0
Ctrl
467-antag
G
Western
WT Liver Cd68 (%)
140
120
100
80
60
Ctrl
467-antag

Figure S7

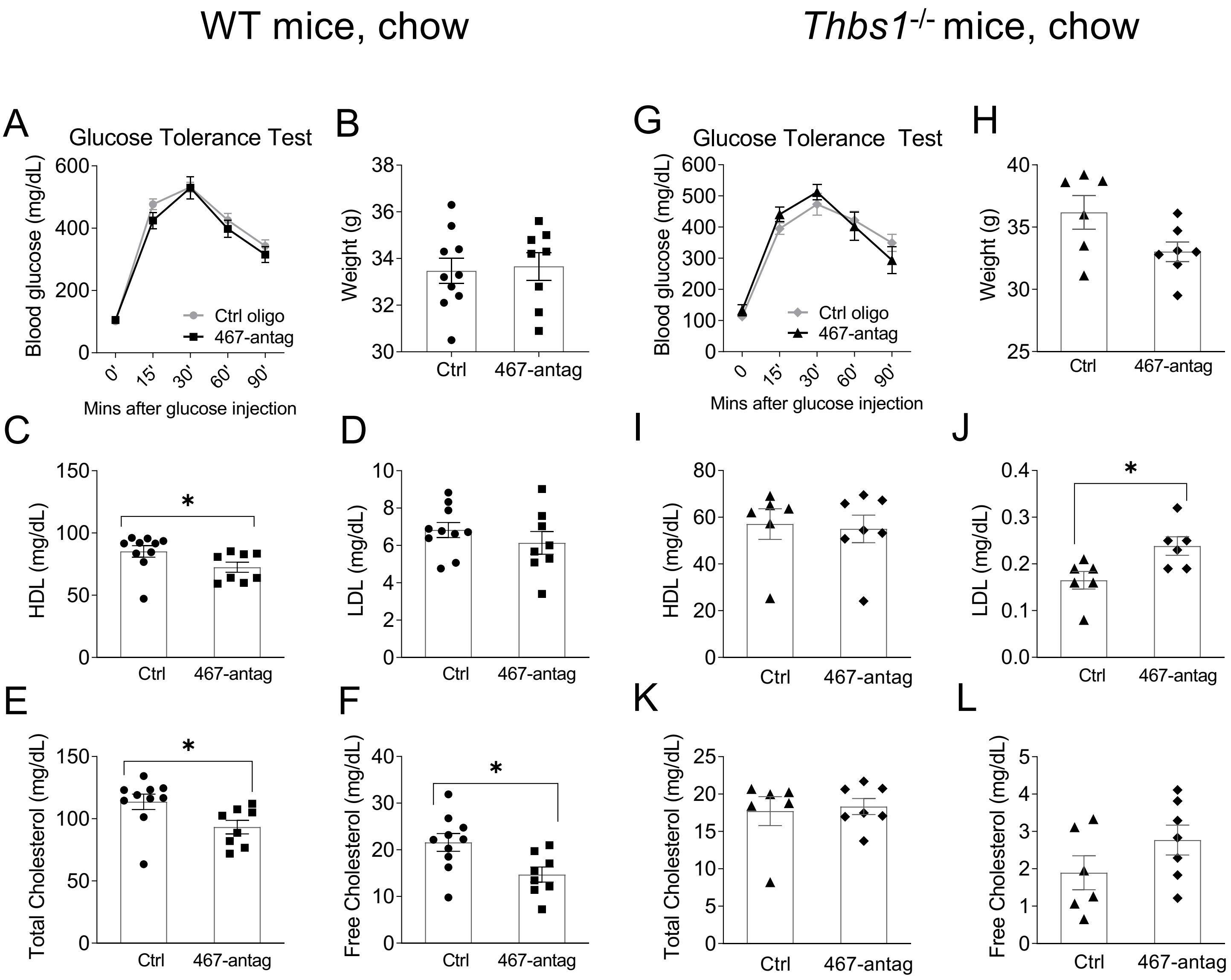

WT mice, chow
Thbs1-/- mice, chow
A
Glucose Tolerance Test
Blood glucose (mg/dL)
Ctrl oligo
467-antag
Mins after glucose injection
B
Weight (g)
Ctrl
467-antag
C
HDL (mg/dL)
D
LDL (mg/dL)
E
Total Cholesterol (mg/dL)
F
Free Cholesterol (mg/dL)
G
Glucose Tolerance Test
H
I
J
K
L

Figure S8

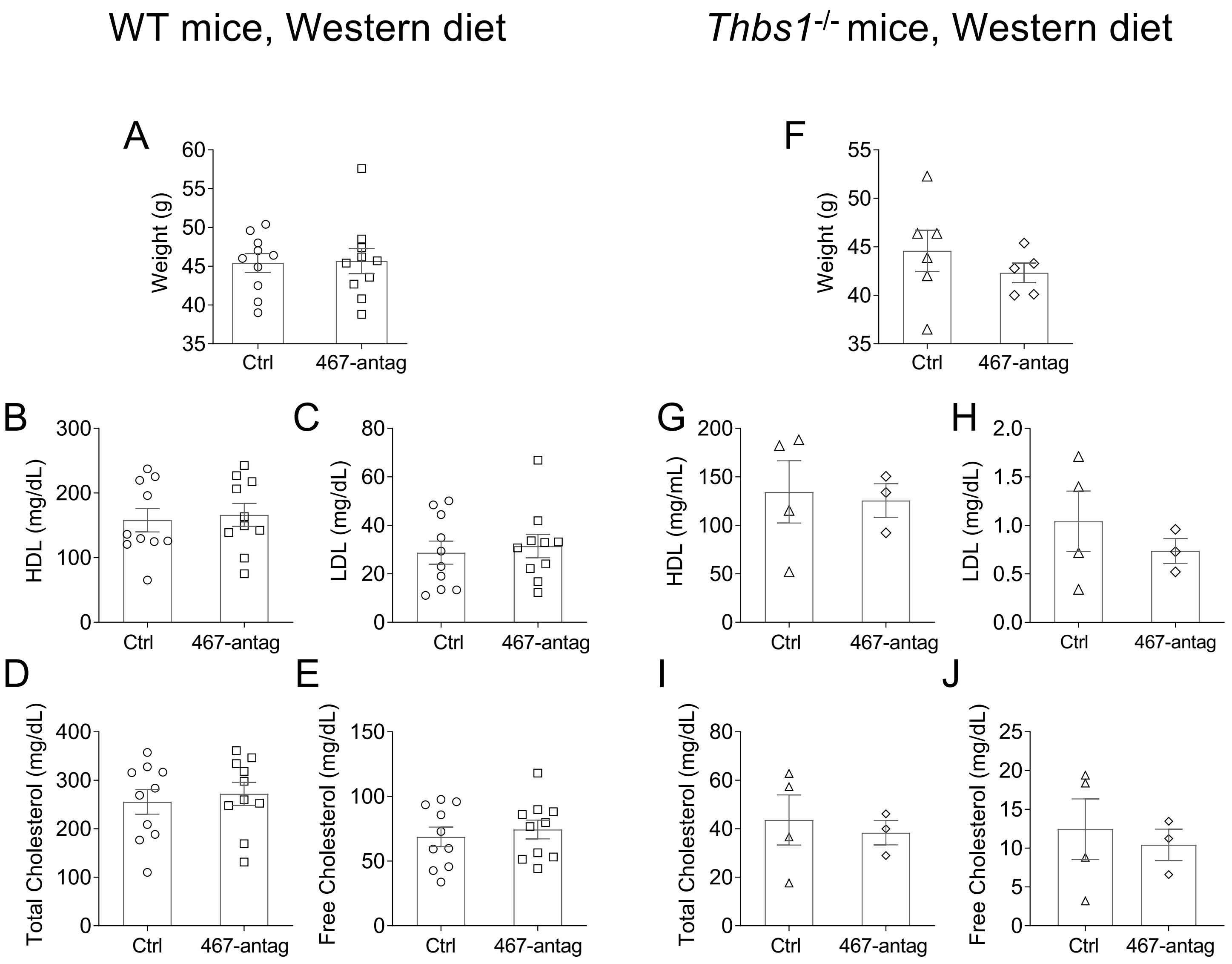

WT mice, Western diet
Thbs1-/- mice, Western diet
A
Weight (g)
60
55
50
45
40
35
Ctrl
467-antag
F
Weight (g)
55
50
45
40
35
Ctrl
467-antag
B
HDL (mg/dL)
300
200
100
0
Ctrl
467-antag
C
LDL (mg/dL)
80
60
40
20
0
Ctrl
467-antag
G
HDL (mg/mL)
200
150
100
50
0
Ctrl
467-antag
H
LDL (mg/dL)
2.0
1.5
1.0
0.5
0.0
Ctrl
467-antag
D
Total Cholesterol (mg/dL)
400
300
200
100
0
Ctrl
467-antag
E
Free Cholesterol (mg/dL)
150
100
50
0
Ctrl
467-antag
I
Total Cholesterol (mg/dL)
80
60
40
20
0
Ctrl
467-antag
J
Free Cholesterol (mg/dL)
25
20
15
10
5
0
Ctrl
467-antag

Figure S9 Major Glucose Transporters

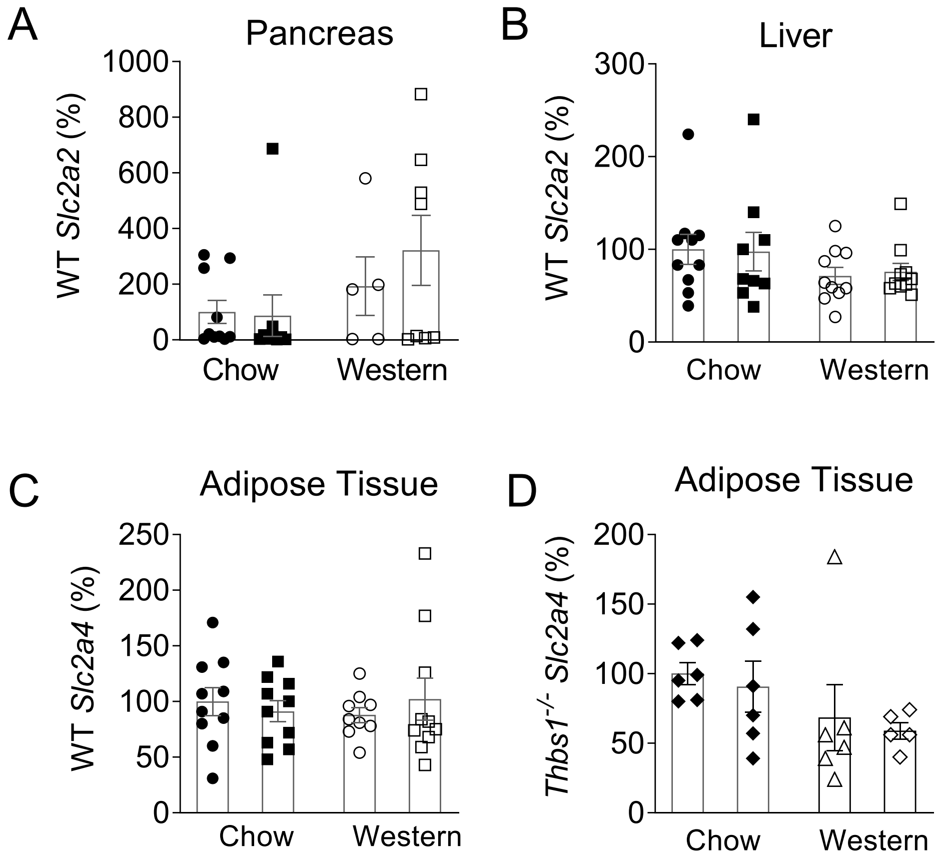

Figure S10

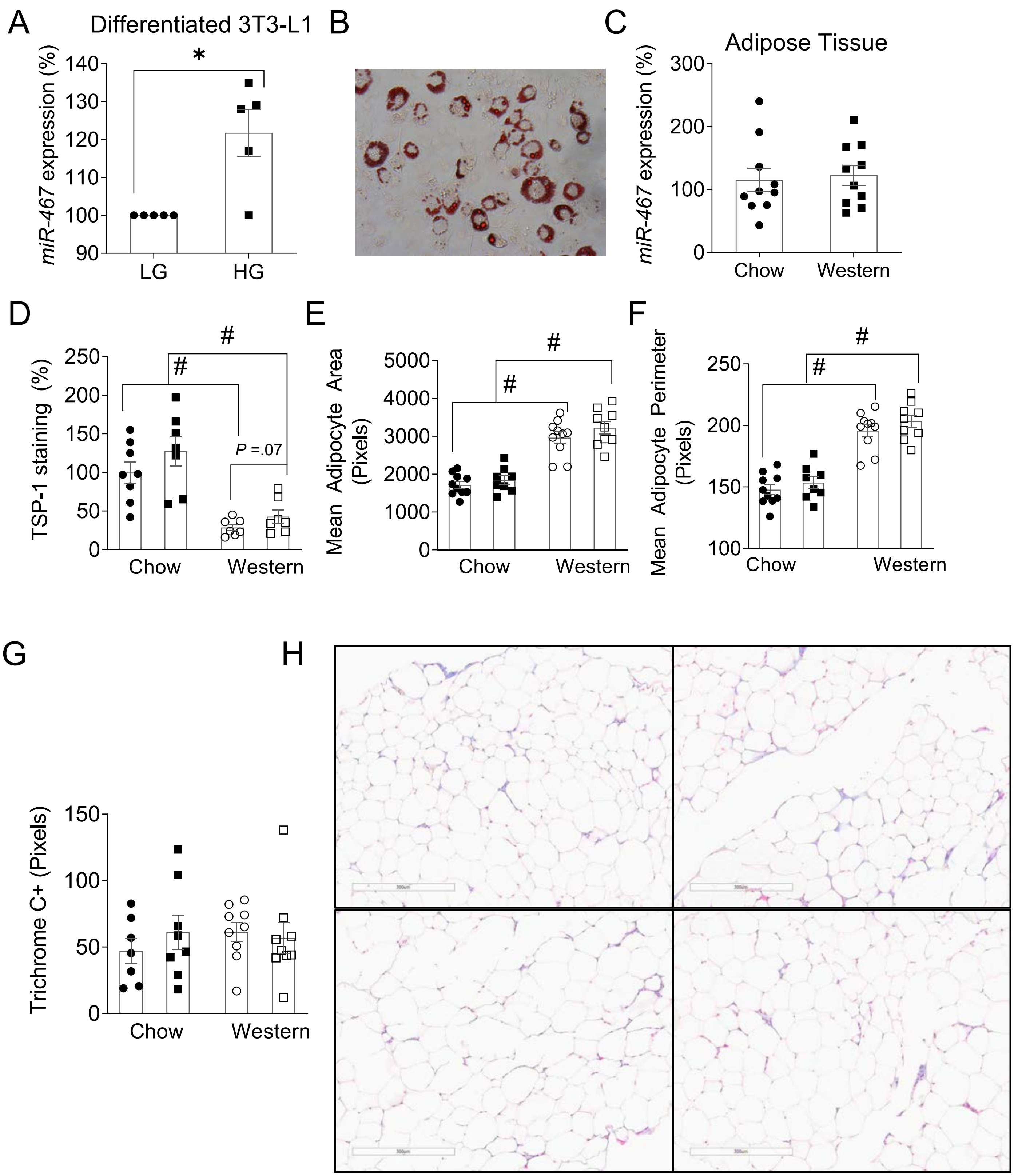
A
Differentiated 3T3-L1
miR-467 expression (%)
140
130
120
110
100
90
*
LG
HG
B
C
Adipose Tissue
miR-467 expression (%)
300
200
100
0
Chow
Western
D
TSP-1 staining (%)
250
200
150
100
50
0
#
#
P =.07
Chow
Western
E
Mean Adipocyte Area (Pixels)
5000
4000
3000
2000
1000
0
#
#
Chow
Western
F
Mean Adipocyte Perimeter (Pixels)
250
200
150
100
#
#
Chow
Western
G
Trichrome C+ (Pixels)
150
100
50
0
Chow
Western
H

Figure S11

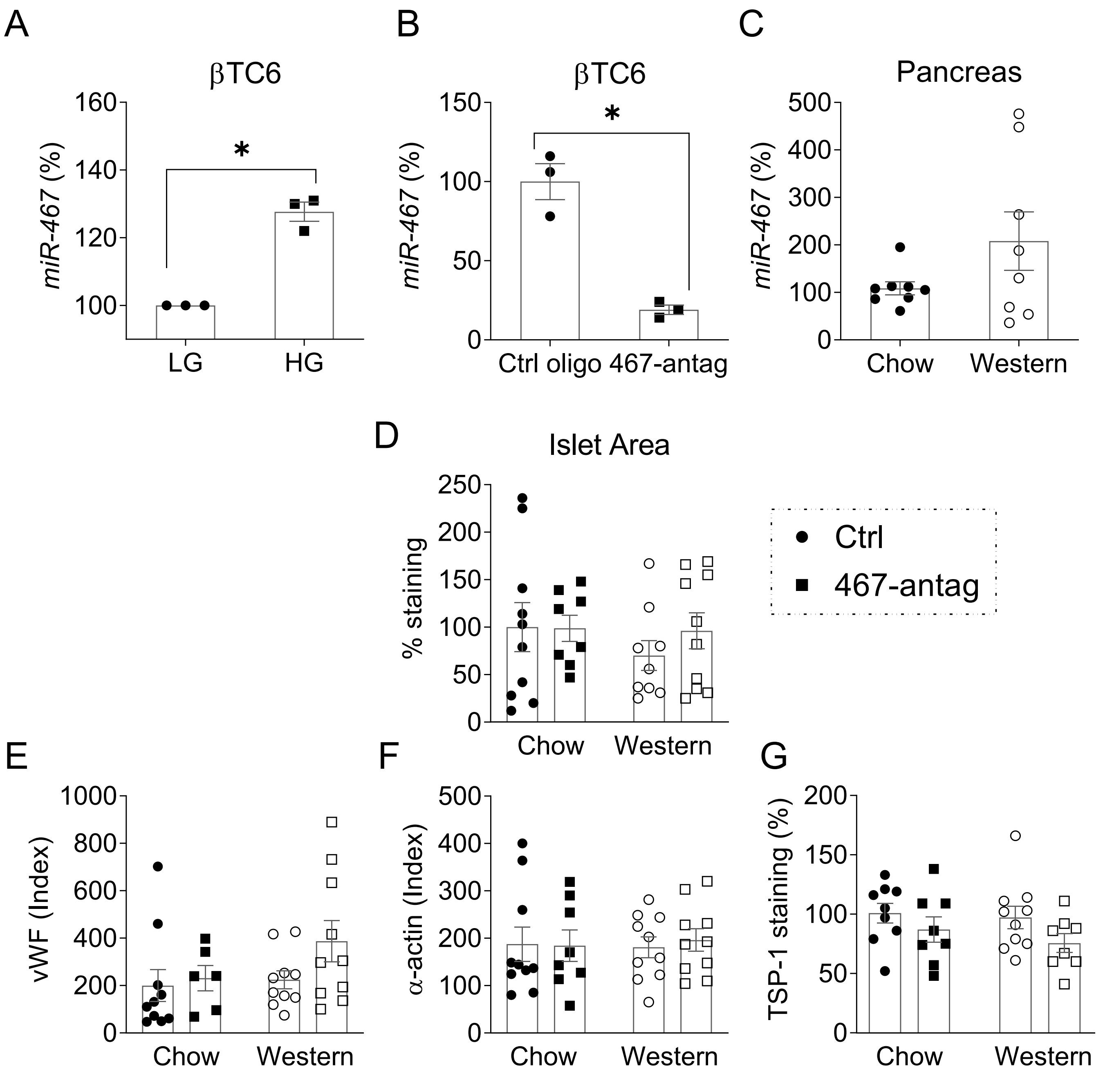
A
βTC6
miR-467 (%)
160
140
120
100
*
LG
HG
B
βTC6
miR-467 (%)
150
100
50
0
*
Ctrl oligo
467-antag
C
Pancreas
miR-467 (%)
500
400
300
200
100
0
Chow
Western
D
Islet Area
% staining
250
200
150
100
50
0
Chow
Western
Ctrl
467-antag
E
vWF (Index)
1000
800
600
400
200
0
Chow
Western
F
α-actin (Index)
500
400
300
200
100
0
Chow
Western
G
TSP-1 staining (%)
200
150
100
50
0
Chow
Western

Figure S12 Gluconeogenesis Genes in WT Liver

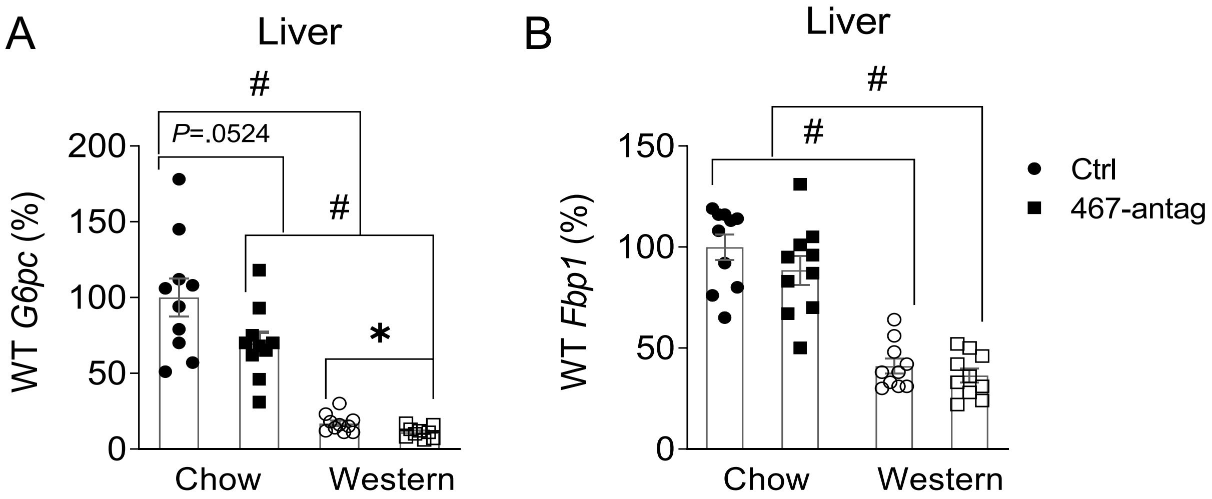

Figure S13

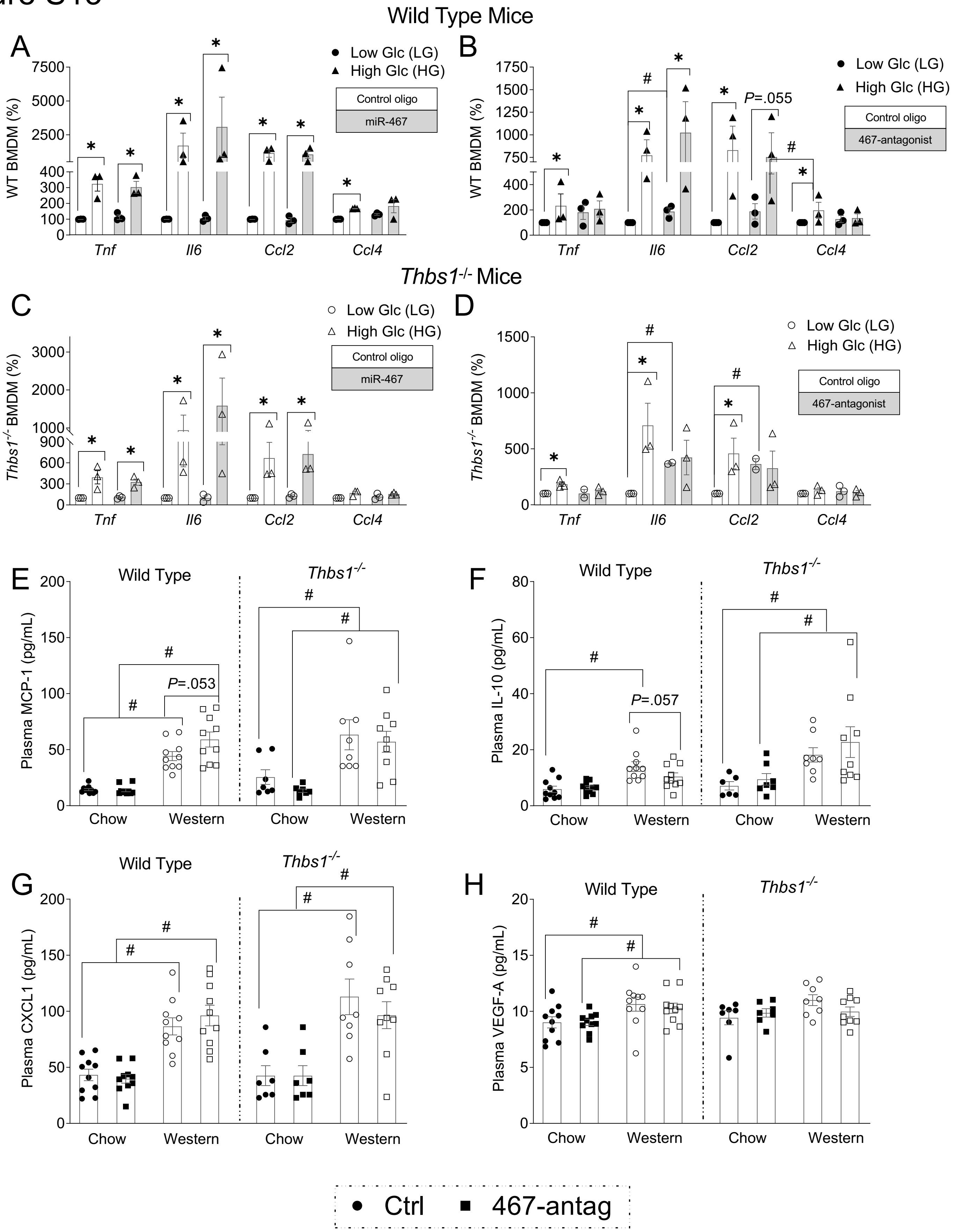

Wild Type Mice
A
Low Glc (LG)
High Glc (HG)
Control oligo
miR-467
WT BMDM (%)
Tnf
Il6
Ccl2
Ccl4
B
Low Glc (LG)
High Glc (HG)
Control oligo
467-antagonist
P=.055
Thbs1-/- Mice
C
Control oligo
miR-467
Thbs1-/- BMDM (%)
D
Control oligo
467-antagonist
E
Wild Type
Thbs1-/-
Plasma MCP-1 (pg/mL)
P=.053
Chow
Western
F
Plasma IL-10 (pg/mL)
P=.057
G
Plasma CXCL1 (pg/mL)
H
Plasma VEGF-A (pg/mL)
Ctrl
467-antag
