# Supplementary material for: miR‐467 regulates inflammation and blood insulin and glucose
Source: J Cell Mol Med. 2021 Feb 10;25(5):2549–62. doi: 10.1111/jcmm.16224 (PMC7933977; doi:10.1111/jcmm.16224)
Supplement: Supplementary file 2 — Supplementary Material [file JCMM-25-2549-s002.docx]

**2. MATERIALS AND METHODS**

**2.1 Experimental animals**

Animal procedures were approved by the Institutional Animal Care and Use Committee. Up to 5 mice were housed per cage and allowed access to food *ad libitum.* Male WT C57BL6 (n=10/group) or *Thbs1^-/-^* (n=7/group) mice were fed a chow or Western diet (TD.88137, 40-45% kcal from fat, 34% sucrose by weight, Envigo) starting at 4 weeks of age and injected weekly with a miR-467a-5p antagonist (2.5 mg/kg body weight) (or a control oligonucleotide that does not have predicted targets in the mouse and human genomes^22^, ^28^), intraperitoneally, starting at 5 weeks of age until the end of the experiment. Body weight was measured weekly.

**2.2 miR-467a-5p mimic and the miR-467a-5p antagonist**

The miR-467a-5p mimic and the control oligonucleotide were purchased from Dharmacon. Cholesterol conjugated miR-467a-5p was modiﬁed by tagging a ﬂuorophore (DY547) and a cholesterol moiety. The custom LNA-modified miR-467a-5p antagonist (TacaTGcaGGcacTTa) and a control oligonucleotide (TTTaGaccgaGcgTGt) were from Qiagen.

**2.3 Glucose and insulin tolerance tests (GTT and ITT)**

GTT and ITT were administered after overnight fasting. Glucose (2 g/kg body weight) or insulin (50 µg/kg) (Sigma) were injected intraperitoneally. Blood glucose levels were measured 0 – 180 min after injections using an AlphaTRAK glucometer. The glucose removal rate (K_itt_), expressed as % / minute, was calculated using the following formula: (0.0693/(t_1/2_) x 100. Plasma glucose (t_1/2_) was calculated from the slope of the least squares curve analysis during the period when plasma glucose concentrations decreased linearly, from 0 – 60 min ^29,30^.

**2.4 Induction of diabetes in mice**

Male mice were injected intraperitoneally with streptozotocin (STZ, 50 mg/kg, Sigma) for 5 consecutive days. Mice with blood glucose >250 mg/dL were selected for experiments.

**2.5 Blood cell counts, HDL/LDL cholesterol, and cytokines in blood**

Blood was collected by cardiac puncture and circulating blood cell counts were analyzed using an ADVIA 120 Hematology System (Siemens). Plasma insulin was measured using Insulin Mouse ELISA kit (Thermo).

A custom U-plex Assay Platform (MSD) was used to assess plasma levels of CCL2 (MCP-1), IL-10, CXCL1, and VEGF-A.

HDL and LDL cholesterol were measured using the HDL and LDL/VLDL quantification kit (BioVision) at end of the experiment.

**2.6 Immunohistochemical staining**

Visceral (omental) adipose tissue and pancreas were fixed in 4% formaldehyde (Electron Microscopy Sciences) for 24 hours, transferred into 70% ethyl alcohol, and embedded in paraffin blocks. Two 5 μM sections of tissue per animal were stained with Hematoxylin (Ricca), Eosin (Protocol), Masson’s trichrome, or specific antibodies. H&E stained sections of adipose tissue were analyzed by ERT Imaging (Cleveland, OH) to determine adipocyte sizes.

Using VECTASTAIN ABC-HRP Kit (Vector Labs), sections were stained with anti-CD68 (biotinylated clone FA-11, 1:10, AbD Serotec), anti-Insulin (1:100, Dako), MOMA-2 (1:25 AbD Serotec), anti-vWF (1:400, Dako), anti-α-actin (clone ab5694 1:200, Abcam), or anti-TSP-1 Ab4 (clone 6.1 1:100, Thermo). Secondary antibodies were included in the species-specific kit and were followed by ImmPACT DAB peroxidase substrate (Vector Labs). Slides were scanned using Leica SCN400 or Aperio AT2 at 20X magnification. Quantification of positive staining was performed using Photoshop CS2 (Adobe) or Image Pro Plus (7.0).

**2.7 Cell culture**

RAW264.7, THP-1, βTC6 and 3T3-L1 cells were purchased from ATCC and cultured according to ATCC directions. THP-1 cells were differentiated in 100 nM PMA (Sigma) for 3 days before glucose stimulation. 3T3-L1 cells were differentiated at 80% confluency with 1µM Dexamethasone, 0.5 mM IBMX, and 1 µg/mL Insulin (all from Sigma).

**2.8 Isolation of bone marrow-derived macrophages (BMDM)**

Bone marrow was collected from femurs and tibia as described in ^31^. Macrophages were differentiated from whole bone marrow using 30 ng/mL MCSF (Biolegend) for 4 days, followed by 15 ng/mL MCSF for 3 days.

**2.9 Glucose stimulation of RAW264.7, differentiated THP-1,** **βTC6, and BMDM**

Up to 1.0 x10^6^ cells were plated in complete media in 6-well plates (Corning). Once glucose levels reached the fasting level (90 mg/dL) as measured using AlphaTRAK glucometer, cells were stimulated with 30 mM D-glucose High Glucose, “HG” (Sigma) for 6 hours (RAW 264.7 and BMDM), 3 hours (3T3-L1) or 30 minutes (βTC6).

**2.10 Transfection of cultured cells**

Transfection of the miR-467a-5p antagonist and its control oligo were aided with Oligofectamine (Invitrogen) for 24 hours. Successful transfection with the cholesterol-modified miR-467a-5p mimic was confirmed by fluorescence 24 hours post-transfection using an inverted microscope DMI6000SD (Leica).

**2.11 Oil Red O Staining**

Differentiated 3T3-L1 cells were washed with 1X PBS and fixed in 10% formalin (Electron Microscopy Sciences) for 15’ at room temperature (RT), washed with 60% isopropanol (Sigma), and stained in the Oil Red O solution for 10’ at RT.

**2.12 RNA Extraction and RT-qPCR**

RNA was isolated using Trizol reagent (Thermo). Organs were flash frozen in liquid nitrogen and homogenized in Trizol. RNA was quantified using Nanodrop 2000 (Thermo).

To measure miR-467a-5p expression, 1 – 2.5 μg of total RNA was first polyadenylated using NCode miRNA First-Strand cDNA Synthesis kit (Invitrogen) or miRNA 1st strand cDNA synthesis kit (Agilent). Real-time qPCR amplification was performed using SYBR GreenER™ qPCR SuperMix Universal (Thermo) or miRNA QPCR Master Mix (Agilent). The miR-467a-5p primer (GTA AGT GCC TAT GTA TATG) was purchased from IDT.

To measure expression of inflammatory markers, 1 – 2 μg of total RNA was used to synthesize cDNA using the SuperScript First-Strand cDNA Synthesis System for RT-PCR (Invitrogen). Real-time qPCR was performed using TaqMan primers for *Tnf*, *Il6*, *Ccl2*, *Il1b*, *Il10*, *Ccl4, Cd68, Slc2a1, Slc2a2, Slc2a4, G6pc, Fbp1* (Thermo) and TaqMan Fast Advanced Master Mix (Thermo). Ct values were determined as described previously ^32^.

β-actin primers (CAT GTA CGT TGC TAT CCA GGC, IDT) were used for normalization by the the 2^−ΔΔCt^ method. All samples were assayed in triplicates using a fluorescence-based, real-time detection method (BioRad MyIQ RT-PCR, Thermo).

**2.13 Statistical analysis**
Data are expressed as the mean value ± Se (standard error). Statistical analysis was performed using GraphPad Prism 5 Software. Student’s t-test and ANOVA were used to determine the significance of parametric data, and Mann-Whitney test was used for nonparametric data. A *P*-value of <.05 was considered statistically significant.

# **Supplementary Figure Legends**

**Figure S1. Representative images of macrophage accumulation markers and tissue structure in WT adipose tissue and pancreas.**

Representative images in WT adipose tissue (A, B) or pancreas (C, D) are shown. Adipose tissue was stained with anti-MOMA-2 antibody (A) or H&E (B). Pancreas was stained with anti-CD68 antibody (C) or H&E (D). Scale bars at 200µM (MOMA-2 IHC adipose tissue scale bars at 100 µM). n=10 mice/group. Arrows show the crown structures.

**Figure S2. Effect of Western diet on macrophage accumulation in adipose tissue and pancreas in WT mice.**

Effects of the Western diet on macrophage accumulation in adipose tissue and pancreas was determined by (A) anti-MOMA-2 or (B) anti-CD68 in WT mice (injected with the control oligonucleotide), respectively. In AT, positive staining was normalized to mean adipocyte area for adipose tissue since adipocyte sizes were changed between groups. n=10 mice/group. Data are normalized to Chow ctrl diet average staining. **P*<.05

**Figure S3. miR-467 antagonist has no effect on circulating monocytes or WBC in WT or *Thbs1*^-/-^ mice.**

Whole blood was collected at end point and analyzed on a hematology analyzer to determine circulating numbers of monocytes and WBCs in WT (A – C) or *Thbs1^-/-^* mice (D – E). # *P*< .05 vs chow diet.

**Figure S4. Adipose tissue inflammation in increased in WT mice on Western diet.**

Effect of Western diet on expression of pro-inflammatory markers (*Il6*, *Tnf*, *Ccl2*, *Ccl4*, *Il1b*) were assessed in whole adipose tissue by RT-qPCR from WT mice injected with control oligonucleotide. Data is normalized to β–actin. Data are relative to Chow ctrl diet average, n=10 mice/group. **P*<.05

**Figure S5. Effect of Western diet on macrophage accumulation in adipose tissue and pancreas in *Thbs1*^-/-^ mice.**Effects of the Western diet on macrophage accumulation in adipose tissue and pancreas was determined by anti-MOMA-2 (A) or anti-CD68 (B) in *Thbs1*^-/-^ mice (injected with the control oligonucleotide), respectively. In AT, positive staining was normalized to mean adipocyte area for adipose tissue since adipocyte sizes were changed between groups. n=7 mice/group. Data are normalized to Chow ctrl diet average staining. **P*<.05

**Figure S6. Effects of miR-467a-5p antagonist on liver.**

RNA from whole liver was extracted at the end of the experiment. Expression of miR-467 (A), inflammatory markers *Tnf* (B, C), *Il1b* (D, E), or macrophage marker *Cd68* (F, G) were assessed in WT mice on chow (B, D, F) or Western diet (C, E, G). Data are relative to ctrl oligo average (to Chow ctrl average in A). n=10 mice/group. **P*<.05 vs ctrl oligo, # *P*<.05 vs chow diet

**Figure S7** **Effects of miR-467a-5p antagonist on mouse weight or blood lipid profile in chow-fed WT or *Thbs1*^-/-^ mice**

Time course for the intraperitoneal glucose tolerance test (GTT) at the end of the experiment in chow-fed WT (A) or *Thbs1*^-/-^ (G) mice. Mouse weight measured at the end of study in WT (B) or *Thbs1*^-/-^ (H) mice. A quantification kit was used to quantify HDL (C, I), LDL (D, J), total cholesterol (E, K) or free cholesterol (F, L) from serum in WT or *Thbs1*^-/-^ mice, respectively. WT: n=10 mice/group. *Thbs1*^-/-^: n=7 mice/group. **P*<.05

**Figure S8** **Effects of miR-467a-5p antagonist on mouse weight or blood lipid profile in WT or *Thbs1*^-/-^ mice on Western diet.**

Mouse weight measured at the end of study in WT (A) or *Thbs1*^-/-^ (F) mice on a Western diet. A quantification kit was used to quantify HDL (B, G), LDL (C, H), total cholesterol (D, I) or free cholesterol (E, J) from serum in WT or *Thbs1*^-/-^ mice, respectively. WT: n=10 mice/group. *Thbs1*^-/-^: n=7 mice/group.

**Figure S9 Expression of major glucose transporters in pancreas, liver, and adipose tissue.**

Expression of the major glucose transporters were measured: *Slc2a2* (Glut2) in pancreas (A) and liver (B) and *Slc2a4* in AT (C, D). Data are normalized to the chow ctrl average.

**Figure S10. miR-467 in adipose tissue and the effects of the miR-467 antagonist injections.**

(A) miR-467 expression was measured 3 hrs post high glucose (HG) stimulation in cultured mouse fibroblasts (3T3-L1) differentiated into adipocytes. LG: low glucose control (5 mM D-glucose). HG: high glucose stimulated (30 mM D-glucose). Data is normalized to LG ctrl average. n=5 independent replicates. **P* <.05. (B) Representative phase contrast image of Oil Red O Staining of 3T3-L1 cells at day 7 post-differentiation. 20x magnification. (C) Expression of miR-467 in WT C57/BL6 mouse adipose tissue on chow or Western diet for 32 weeks. n=10 mice/group. (D) Quantification of the % positive staining with an anti-TSP-1 antibody. (E) Mean adipocyte area and perimeter (F) were quantified from H&E-stained sections of adipose tissue. (G) Quantification of blue color density from Masson’s trichrome staining. (H) Representative images of trichrome staining. Scale bars at 300 µM. # *P*<.05 vs chow diet.

**Figure S11.** **miR-467 in pancreas and the effects of the miR-467 antagonist injections.**

(A) miR-467 expression was measured 30’ post HG stimulation in a cultured mouse β cell line (βTC6). Data is normalized to LG ctrl average. n=3 independent replicates. LG: low glucose control (5 mM D-glucose). HG: high glucose stimulated (30 mM D-glucose). **P*<.05 (B) Expression of miR-467 in C57/BL6 WT mouse pancreas on chow or Western diet for 32 weeks. n=10 mice/group. (C) Pancreas sections were stained for insulin and counterstained with hematoxylin. Islet area was quantified as % positive insulin staining over the total area per 100 pixels. Quantification of positive staining in pancreas using an anti-vWF (D), anti-α-actin, (E) or anti-TSP-1 (F) antibody.

**Figure S12. Gluconeogenesis genes in liver from WT mice.**

Expression of the gluconeogenesis genes, *G6pc* (A) or *Fbp1* (B) in liver were assessed in WT mice. Data are normalized to the chow ctrl average. n=10 mice/group. # *P*<.05 vs chow diet.

**Figure S13. miR-467 blocks pro-inflammatory functions of cultured macrophages.**

Effect of miR-467 or miR-467 antagonist on expression of pro-inflammatory markers (*Tnf*, *Il6*, *Ccl2*, and *Ccl4*) were assessed in BMDM from (A, B) WT or (C, D) *Thbs1*^-/-^ mice transiently transfected with miR-467 (A, C) or a miR-467 antagonist (B, D) compared to a control oligo. RNA was collected 6 hrs post glucose stimulation by RT-qPCR and normalized to β–actin. Data are relative to the LG control stimulated samples per transfection, n=3 independent replicates. BMDM: bone marrow-derived macrophages. LG: low glucose control (5 mM D-glucose). HG: high glucose stimulated (30 mM D-glucose). **P* <.05 compared to LG ctrl. # *P* <.05 vs chow diet. (E – H) Plasma from WT or *Thbs1^-/-^* mice, collected at 32 weeks, were assayed by U-plex for circulating (E) MCP-1, (F) IL-10, (G) CXCL1 and (H) VEGF-A. # *P* <.05 vs chow diet.
